# Fast, simple, and informative patient-specific dose verification method for intensity modulated total marrow irradiation with helical tomotherapy

**DOI:** 10.1186/1748-717X-9-34

**Published:** 2014-01-25

**Authors:** Yutaka Takahashi, Susanta K Hui

**Affiliations:** 1 Masonic Cancer Center, University of Minnesota, 424 Harvard Street SE, Minneapolis 55455, MN, USA; 2 Department of Therapeutic Radiology, University of Minnesota, 424 Harvard Street SE, Minneapolis 55455, MN, USA

## Abstract

**Background:**

Patient-specific dose verification for treatment planning in helical tomotherapy is routinely performed using a homogeneous virtual water cylindrical phantom of 30 cm diameter and 18 cm length (Cheese phantom). Because of this small length, treatment with total marrow irradiation (TMI) requires multiple deliveries of the dose verification procedures to cover a wide range of the target volumes, which significantly prolongs the dose verification process. We propose a fast, simple, and informative patient-specific dose verification method which reduce dose verification time for TMI with helical tomotherapy.

**Methods:**

We constructed a two-step solid water slab phantom (length 110 cm, height 8 cm, and two-step width of 30 cm and 15 cm), termed the Whole Body Phantom (WB phantom). Three ionization chambers and three EDR-2 films can be inserted to cover extended field TMI treatment delivery. Three TMI treatment plans were conducted with a TomoTherapy HiArt Planning Station and verified using the WB phantom with ion chambers and films. Three regions simulating the head and neck, thorax, and pelvis were covered in a single treatment delivery. The results were compared to those with the cheese phantom supplied by Accuray, Inc. following three treatment deliveries to cover the body from head to pelvis.

**Results:**

Use of the WB phantom provided point doses or dose distributions from head and neck to femur in a single treatment delivery of TMI. Patient-specific dose verification with the WB phantom was 62% faster than with the cheese phantom. The average pass rate in gamma analysis with the criteria of a 3-mm distance-to-agreement and 3% dose differences was 94% ± 2% for the three TMI treatment plans. The differences in pass rates between the WB and cheese phantoms at the upper thorax to abdomen regions were within 2%. The calculated dose agreed with the measured dose within 3% for all points in all five cases in both the WB and cheese phantoms.

**Conclusions:**

Our dose verification method with the WB phantom provides simple and rapid quality assurance without limiting dose verification information in total marrow irradiation with helical tomotherapy.

## Background

Helical tomotherapy offers a high intensity modulated beam with multileaf collimator while translating the couch into the gantry [1,2]. This allows the delivery of an intensity modulated beam for the treatment of very long targets, such as in total body irradiation (TBI) and total marrow irradiation (TMI) [3-11].

To check the accuracy of dose calculations and mechanical issues radiation therapy treatment planning, each intensity modulated treatment plan requires dose verification before the treatment delivery to the patient. Currently, each individual plan is calculated in a phantom geometry and delivered to the phantom. The dose is then measured with ion chambers and radiographic or radiochromic films, or with detector arrays alone [12,13]. The calculated dose is then compared with the measured dose. This procedure is called delivery quality assurance (DQA). Most users currently perform DQA using the cylindrical Virtual Water™ solid water phantom, which was designed and provide by Accuray, Inc with a 30 cm diameter and 18 cm length (Cheese phantom). This phantom accepts ion chambers and a radiographic or a GafChromic film in the coronal or sagittal plane.

Unlike treatment of solid tumors, including head and neck, lung, and prostate cancers, the treatment length and time of TBI or TMI are markedly long, at more than 90 cm and 30 minutes, respectively [5-7,9]. The complexities are compounded as there are many organs at risk including the brain, lung, liver, kidneys, and peritoneal cavity as well as targets (marrow) throughout the whole body in TMI. In addition, a number of tomotherapy machines still do not have a dose servo system. These machines show output fluctuation [13-17], particularly in long treatment deliveries, corresponding with the later part of the treatment site (i.e., pelvis and femur) in TMI. These facts highlight the importance of dose verification for a wide range of targets where there are critical organs around to assure the safety of TMI patients. Since the cheese phantom is only 18 cm long, single delivery of DQA in TMI covers only a small portion of the target. Complete verification from the head to femur regions requires repeating the DQA procedure at least three times for an adult TMI case, leading to a significant increase in DQA time.

Another possible method of verifying dose distribution is to use 2D or 3D detector arrays. Although these have dramatically sped up IMRT dose verification, commercially available arrays are not suitable in TMI because of their limited array size, which requires multiple DQA delivery. Furthermore, the electronic circuit of a 2D or 3D array will be irradiated in TMI delivery, resulting in damage to the detector array. The only way to verify the dose and dose distribution in such situations is to use film.

Here, we propose a simple, fast and informative DQA method for TMI delivery with film.

## Methods

1. Set up of the cheese phantom, ion chambers and films.

**Figure 1:**
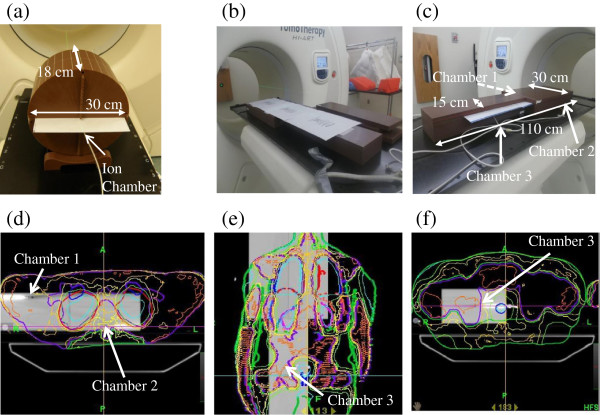
**Scheme of DQA in TMI planning. (a)** Scheme of DQA with the cheese phantom **(b)** Film alignment in the WB phantom, **(c)** ion chamber alignment in the WB phantom. The WB phantom was moved so that the three ion chambers were located at approximately **(d)** the upper humerus, **(e)** thoracic vertebrae and **(f)** pelvic bone in the dose verification, representing locations where bone marrow is abundant. These regions are also suitable for point dose measurement because of a high dose and low dose gradient.

The cheese phantom made of Virtual water™ is a cylindrical phantom of 15 cm radius and 18 cm length which is cut into two semi cylindrical halves where a sheet of film can lie along the central axis of the phantom (Figure 1 (a)). The phantom also has a series of holes to allow for the insertion of an Exradin A1SL ion chamber with the volume of 0.053 cc (A1SL, Standard imaging, WI, USA). In the present study, an ion chamber and an EDR2 film with 30.5 cm length and 25.4 cm width (Kodak, Nagano, Japan) in the coronal plane were placed as shown in Figure 1 (a).

2. Set up of the WB phantom, ion chambers and films

Using similar materials as the cheese phantom, we built a two-step solid water slab phantom as seen in Figures 1 (b) and (c). This Whole Body Phantom (WB phantom) offers a total length of 110 cm, 8 cm height and two step widths of 30 cm and 15 cm. Three EDR2 films that cover approximately 90 cm can be inserted into this phantom (Figure 1 (b)) as well as three A1SL ionization chambers. Point doses can then be measured at locations corresponding to the upper humerus (Chamber 1 in Figure 1 (d)), thoracic vertebrae (Chamber 2 in Figure 1 (d)), and pelvic bones (Chamber 3 in Figures 1 (e) and (f)).

3. Treatment planning

TMI treatment planning for five patients was conducted with the TomoTherapy HiArt Planning Station (Accuray, Inc., Madison, WI). Target and avoidance structures including brain, lenses, eyes, parotid glands, thyroids, lungs, heart, kidneys, liver, peritoneum, bladder, and rectum were contoured on a Pinnacle treatment planning system (Philips Medical Systems, Palo Alto, CA). The planning target volume (PTV) was generated by adding margins of 5 mm to thoracic bones, 1.5 cm to femur and shoulder bones, and 1 cm to all other bones. The DICOM-RT structure set was then transferred to the TomoTherapy HiArt Planning Station.

A prescription of 18 Gy/3 fractions was used for planning simulation to cover 85% of PTV with the prescription dose. The pitch, modulation factor and Jaw size were 0.200 or 0.287, 2.5, and 5 cm, respectively. Final dose calculation was performed with the convolution/superposition algorithm with the calculation grid size of 0.391 cm.

4. Delivery QA and the verification analysis

Kilovoltage CT images of the cheese and WB phantoms were taken with the same technique as the TMI patient scans. Five TMI treatment plans were transferred to the tomotherapy DQA workstation.

For DQAs with the cheese phantom, three DQA procedures per patient were created in the Tomo DQA workstation. The cheese phantom was moved three times so that the phantom covered locations corresponding to the head and neck, thorax, or pelvis. The doses were then recalculated in the phantom by using the sinogram from the original TMI planning in human body. This recalculation was based on the image value-to-density calibration (IVDC) table consisting of 13 different density plugs which was also used for the patient dose calculation. These three DQA treatment procedures were delivered to the cheese phantom as a conventional method.

For DQAs with the WB phantom, a single DQA procedure per patient was created in the Tomo DQA workstation. The WB phantom was placed so that the three ion chambers were located at approximately the upper humerus (Figure 1 (d)), thoracic vertebrae (Figure 1 (d)), and pelvic bone (Figures 1 (e), and (f)); representing locations where bone marrow is abundant. These regions are also suitable for point dose measurement because of a high dose and low dose gradient. The three films were placed at the regions of the head and neck to upper thorax, lower thorax to abdomen, and pelvis to femur to include the critical organs such as the brain, lung, kidney, and liver as well as the PTV. The dose was recalculated in the phantom by using the sinogram of the original TMI planning of the human body as shown Figures 1 (d), (e), and (f). The DQA procedure was then delivered to the WB phantom and point doses and planer dose distributions were measured by the ionization chambers and EDR-2 films, respectively. The main difference between DQA deliveries with the cheese phantom and those with the WB phantom was the DQA delivery time, namely three deliveries versus a single delivery.

To analyze differences between measured and calculated doses, the delivered dose distributions need to be registered relative to the phantom. To do that, four points were identified using the fixed cross laser (green laser). Green cross lasers were marked on the film at a thorax-to-abdomen site. For head and neck and pelvic regions, we identified the points at a well-defined distance (e.g.; 20 cm or 25 cm) from the green laser by using a ruler with the aid of the tomotherapy couch coordinate readout.

After irradiation, EDR2 films were developed and scanned using a VIDAR VXR-16 Dosimetry Pro Scanner (VIDAR Systems Corporation, Herndon, VA, USA). The EDR2 film analysis was performed in the DQA workstation. Although Yeo et al. reported that EDR2 film exhibits considerable energy dependence (a maximum discrepancy of 9%, compared with an ion chamber) at 10 cm depth for IMRT field [18], several reports demonstrated EDR2 film could be used for absolute dose measurement with ≤3% uncertainty [19,20]. However, we analyzed the EDR2 films for the relative dose as already published here [13,21-23] to avoid the uncertainty from a film developer. The comparison of longitudinal profiles and gamma analyses with the criteria of 3 mm/3% between planned and measured dose was performed. In the present study, the dose difference criteria in gamma analysis were defined in respect to the prescribed dose.

5. Statistical analysis

The dosimetric results with films and ion chambers between the cheese phantom and the WB phantom as well as the differences in time required in each DQA process were statistically evaluated by Mann–Whitney *U* test using Dr. SPSS II software (IBM, New York, NY).

## Results and discussion

Dose verification is an essential part of IMRT clinical practice. The many organs at risk throughout the whole body and output fluctuation in long beam-on time demand a wide range of dose verifications particularly from head to pelvis where there are critical organs around to ensure patient safety. Currently, most clinics use the cheese phantom for DQA in tomotherapy planning. Since the longitudinal size of this phantom is only 18 cm, the TMI DQA procedure must be repeated at least three times to verify the dose for the head and neck, thoracic and pelvic regions in adult patients, which significantly prolongs the dose verification process.

Here, we developed a simple, fast and informative DQA method in helical tomotherapy for extremely long target treatments. The WB phantom provided point doses and dose distributions from head and neck to femur in a single TMI treatment delivery.

Table 1 shows the average time required in each process in the DQA procedure for five TMI cases in our proposed method (WB phantom) and the standard method (cheese phantom). Our method reduced DQA time by 12 minutes (p < 0.0001), 6 minutes (p < 0.0001), and 76 minutes (p < 0.0001) in the DQA setup, phantom setup and beam delivery, respectively. This decrease is because our method requires only a single delivery to obtain dose distributions from head to pelvis, thus reducing time throughout the entire DQA process.

**Table 1:** Time required in each process in the DQA procedure for five TMI case

	**WB phantom (minute)**	**Cheese phantom (minute)**	**p-value**
DQA setup in the DQA work station	10.4 ± 1.5	22.2 ± 0.8	p < 0.0001
Phantom setup	9.2 ± 1.3	15.0 ± 1.0	p < 0.0001
Beam delivery	37.8 ± 2.3	113.4 ± 6.9	p < 0.0001
Total	57.4 ± 2.3	150.6 ± 6.1	p < 0.0001

**Figure 2:**
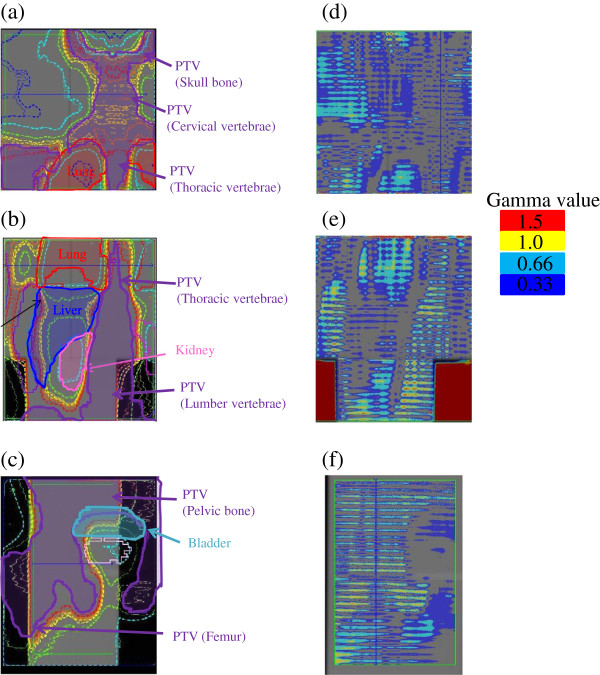
**Comparison of dose distributions in TMI planning between calculated and measured doses by three EDR-2 films in the WB phantom. (a)** The isodose lines were overlaid at **(a)** head and neck to upper thorax region, **(b)** lower thorax to abdominal region, and **(c)** pelvic bone to femur region, **(d)** gamma distribution in the region shown in **(a)**, **(e)** gamma distribution in the region shown in **(b)**, **(f)** a gamma distribution in the region shown in **(c)**. Note that gamma value outside the phantom as shown in figure **(e)** was not included to calculate the pass rate.

Figure 2 shows a representative dose verification case using the WB phantom with EDR2 films. Good agreement of calculated and measured doses was found at the neck to upper thorax regions (Figures 2 (a), (d)), lower thorax to abdomen regions (Figures 2 (b), (e)), and pelvic to femur regions (Figures 2 (c), (f)). The pass rates in gamma analysis with a 3 mm distance-to-agreement and 3% dose differences were 96.8% ± 2.1%, 93.6% ± 0.5%, and 91.6% ± 0.2% for the regions of head and neck–upper thorax, lower thorax-abdomen, and pelvis-femur, respectively (Table 2). Moreover, our results showed that our DQA method could cover the dose distribution from head and neck to femur with the single delivery of a TMI QA procedure.

**Table 2:** **Percentage of points passing gamma criteria of 3%/3** mm **in DQA with EDR2 films for five TMI cases**

**Region**	**WB phantom**	**Cheese phantom**	**P-value**
	**(%)**	**(%)**	
Head and neck to upper thorax	96.8 ± 2.1	95.2 ± 3.1	0.421
Lower thorax to abdomen	93.6 ± 0.5	94.1 ± 2.4	0.548
Pelvis to femur	91.6 ± 0.2	93.5 ± 3.0	0.222

Since use of the cheese phantom is the standard method for DQA, we conducted DQA procedures for three anatomical regions in five TMI treatment plans using both the WB and cheese phantoms. Table 2 compares pass rates in gamma analysis with the same acceptance criteria for the five TMI cases between the two phantoms. The differences in pass rate between cheese phantom and WB phantom were within 3% with no statistical significance for five TMI cases. The worst pass rate was 90% at the pelvic-femur region with both phantoms, but this is considered acceptable according to the report of AAPM task group 148 [13].

Table 3 shows the results of point dose verification with ion chambers for the five TMI cases. The calculated dose agreed with the measured dose within 3% for all points in all five cases with both the WB and cheese phantom. These results indicate that the WB phantom provides closely similar results to the cheese phantom.

**Table 3:** Agreement of calculated dose with ion chamber measurements in% for five TMI cases

**Region**	**WB phantom**	**Cheese phantom**	**P-value**
	**(%)**	**(%)**	
Head and neck to upper thorax	−0.14 ± 1.44	1.36 ± 1.19	0.151
Lower thorax to abdomen	0.26 ± 1.8	0.97 ± 1.41	0.548
Pelvis to femur	0.31 ± 2.52	0.41 ± 1.39	0.222

Dose verification for TBI or TMI with helical tomotherapy has been performed with various phantoms and detectors. Gruen et al. performed dose verification using a cheese phantom for point dose measurement with ion chambers [4]. Zhuang et al. used a Meditec solid water phantom of 30 cm (length) × 30 cm (width) × 12 cm (depth) with a 0.6 cc PTV farmer-type ion chamber and EDR2 film [24]. Hui et al. used a cylindrical phantom of 30 cm diameter and 36 cm length [7]. None of these are long enough to verify the large treatment lengths used in TBI or TMI. In addition, use of a 0.6 cc ion chamber is not always appropriate in verification of high intensity modulated plans because of the large size of the ion chamber. Several groups have used TLDs in a rando phantom to verify doses for TMI or TBI, [4,6,25]; this allows measurement of point doses in the whole body in a single delivery of a TMI treatment plan. In our method, one delivery of the DQA procedure enables the measurement of both point doses using smaller ion chambers (0.053 cc compared to 0.6 cc farmer type ion chamber) and dose distributions from the head and neck to pelvis, which offers more informative QA results.

The advantages of our DQA method are not only a reduction in DQA time and coverage of dose distribution from the head and neck to femur, but also a decrease in cost, since it uses a vendor-supplied phantom and the detectors present in a tomotherapy machine.

The number of clinics providing TBI or TMI with helical tomotherapy in the past few years has increased [3,5,7-11,24]. Dosimetric studies on volumetric arc therapy for TBI or TMI with conventional linear accelerators have also been extensively performed [25-28]. These studies used an electric portal imaging system, or 2D or 3D diode arrays [25-28]. Although these devices offer fast and reliable dose verification for majority of treatment, commercially available 2D and 3D arrays still require multiple treatment delivery in TMI DQA because of the limitation in array size, which significantly prolong the DQA process. Development of a detector array suitable for long treatment deliveries such as TMI would be useful. In the present study, we used EDR2 film to verify 2D dose distribution. Although many clinics have decommissioned their radiographic film developer, EDR2 film has been commonly used in many clinics [17,19-23,29-32]. Currently, an increasing number of clinic use GafChromic films, which do not require a film developer for IMRT QA. Our method can be used for TMI DQA with both GafChromic and EDR2 film.

In addition to its use in tomotherapy, our method is also applicable to long treatments such as TBI, total skin irradiation, and cranial-spinal irradiation with volumetric arc therapy in a conventional linear accelerator, particularly to verify the dose in the field junction.

## Conclusions

Our DQA method with WB phantom could provide a fast, accurate, simple and informative procedure for total marrow irradiation with helical tomotherapy. This technique might offer rapid quality assurance without limiting dose verification information.

## Competing interests

The authors declare that they have no competing interest.

## Authors’ contributions

YT conceived of this study, carried out all the experiments and wrote the article. SH participated in its design and coordination and helped to draft the manuscript. Both authors read and approved the final manuscript.
